# Standardizing persistent and chronic critical illness: impact of definitions variability on prevalence and mortality

**DOI:** 10.1186/s13054-025-05335-5

**Published:** 2025-03-07

**Authors:** Toshinobu Nakai, Yuki Kotani, Yoshiro Hayashi

**Affiliations:** Department of Intensive Care Medicine, Kameda Medical Center, 929 Higashi-Cho, Kamogawa, Chiba 296-8602 Japan

Standardization of terminology and definitions is essential for scientific communication. Without such standardization, some studies may use different terms to express similar conditions, and other studies may use the same term with different definitions. Such diversities in medical language creates inconsistencies in scientific reporting, thereby hindering us from properly understanding the condition.

In this regard, persistent critical illness (PerCI) and chronic critical illness (CCI) are two terms used to describe prolonged critical conditions beyond the acute phase [1, 2]. However, the absence of standardized definitions leads to substantial variability in their clinical implications. Recently, *Critical Care* published a systematic scoping review highlighting the heterogeneity in definitions, epidemiology, and outcomes of PerCI and CCI [3]. We commend the authors for their comprehensive analysis, which synthesizes data obtained from numerous studies, performs a meta-analysis on specific patient populations (e.g., overall populations, sepsis, trauma, and COVID-19), and offers valuable recommendations for future research.

To further expand on the insights provided by Ohbe et al., we conducted an exploratory analysis to illustrate how different PerCI/CCI definitions impact reported prevalence and in-hospital mortality. From the 99 studies included in Ohbe et al.’s scoping review [3], we selected those explicitly reporting PerCI/CCI definitions, prevalence, and in-hospital mortality. We then created a scatter plot, where each dot represents a study, with PerCI/CCI prevalence on the x-axis and in-hospital mortality on the y-axis (Fig. 1), using Excel version 16.94. Dots were color-coded according to the PerCI/CCI definition applied in each study. This visualization underscores the substantial variability in prevalence and mortality based on the chosen definition.

**Fig. 1:**
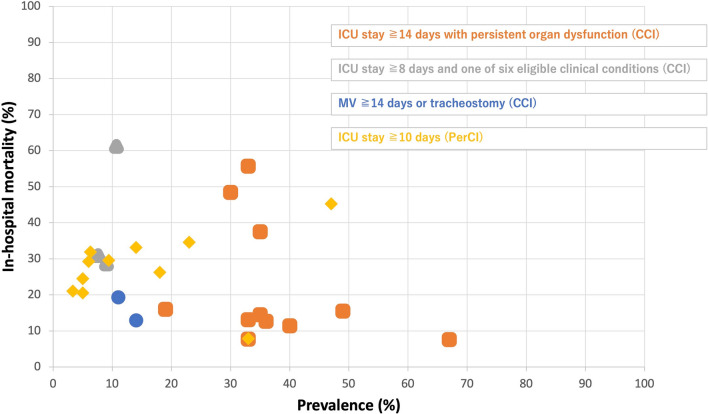
Prevalence and hospital mortality according to the different persistent/chronic critical illness definitions

Studies defining PerCI/CCI as “ICU stay ≥ 14 days with persistent organ dysfunction” (orange) reported higher prevalence compared to other definitions. This trend may be attributed to the study populations, which predominantly consisted of septic patients. Given that sepsis is strongly associated with organ dysfunction and prolonged ICU stays [4], it is reasonable to expect a higher prevalence of PerCI/CCI. Conversely, studies employing the definition “ICU stay ≥ 10 days” (yellow) primarily included non-specific ICU patients (53.3%), resulting in a lower prevalence.

Studies using the definitions “ICU stay ≥ 8 days and one of six eligible clinical conditions" (gray) reported lower prevalence but higher in-hospital mortality. Among these six clinical conditions, prolonged mechanical ventilation was the most common [5], likely selecting a smaller but more critically ill patient group with worse outcomes.

Meanwhile, studies defining PerCI/CCI as “mechanical ventilation ≥ 14 days or tracheostomy” (blue) showed lower prevalence and lower in-hospital mortality. These study populations were predominantly composed of trauma patients, suggesting that the lower mortality rate reflects the relatively favorable prognosis of trauma patients, who typically have fewer comorbidities compared to septic patients.

Our exploratory analysis highlights the pressing need for a standardized PerCI/CCI definition. Establishing a uniform definition is essential for accurate identification, risk stratification, targeted interventions, and shared decision-making. We urge multidisciplinary discussions to optimize long-term care strategies for critically ill patients.

## Data Availability

Further information on the original manuscript is available from the corresponding authors upon reasonable request.

## Abbreviations

PerCI: Persistent critical illness
CCI: Chronic critical illness
ICU: Intensive care unit
